# Loneliness and Cognitive Function in Older Adults Living in Latin America: A Systematic Review

**DOI:** 10.1093/geroni/igaf122.1713

**Published:** 2025-12-31

**Authors:** David Camacho, Pamela Vega, Fernando Wagner, Carolina Santamaria-Ulloa, Amanda Lehning, Joe Gallo

**Affiliations:** University of Illinois Chicago, Chicago, Illinois, United States; Instituto Nacional de Geriatría, Mexico City, Distrito Federal, Mexico; University of Maryland, Baltimore County, Baltimore, Maryland, United States; University of Costa Rica, San Pedro de Montes de Oca, San Jose, Costa Rica; University of Maryland Baltimore, Baltimore, Maryland, United States; Johns Hopkins University, Baltimore, Maryland, United States

## Abstract

Extant systematic reviews with samples from high-income countries have found an inverse relationship between loneliness and cognitive function. Considering that cultural and contextual resources influence the experience of loneliness and cognitive health, we conducted a systematic review analyzing quantitative studies exploring the relationship between loneliness and cognitive function in older adults in Latin America. Following PRISMA guidelines, we used five databases (PubMed, PsycInfo, Scopus, LILACS, and SciELO). Inclusion criteria were: a) quantitative research examining the relationship between loneliness and cognitive health, b) descriptions of loneliness and measures of cognitive function, c) English or Spanish language peer-reviewed articles, and d) a sample of older adults in Latin America (≥60 years). We assessed bias using the Risk of Bias Instrument for Cross-Sectional Surveys of Attitudes and Practices. Seven of the 1,887 studies (all cross-sectional) met the inclusion criteria, comprising 26,440 participants from Brazil or Mexico. Most, but not all, found a significant inverse association between loneliness and cognitive function after controlling for salient health and psychosocial factors. Measures and conceptualizations of loneliness and cognitive function, as well as theoretical explanations linking these concepts, varied. Two studies had a high risk of bias. Current evidence suggests a possible cross-sectional association between loneliness and cognitive function in older adults in these countries. Further research is needed to examine the possible bidirectional relationship using representative samples and longitudinal designs; test pathways linking dimensions of loneliness (e.g., chronicity) to cognitive function (e.g., Alzheimer’s disease continuum), and explore Latin American diversity (e.g., countries, indigenous peoples, sexual minorities).

